# Supplementary material for: Comparison of three common nutritional screening tools with the new European Society for Clinical Nutrition and Metabolism (ESPEN) criteria for malnutrition among patients with geriatric gastrointestinal cancer: a prospective study in China
Source: BMJ Open. 2018 Apr 12;8(4):e019750. doi: 10.1136/bmjopen-2017-019750 (PMC5898289; doi:10.1136/bmjopen-2017-019750)
Supplement: Author's manuscript [file bmjopen-2017-019750.draft_revisions.pdf]

BMJ Open

BMJ Open is committed to open peer review. As part of this commitment we make the peer review history of every article we publish publicly available.

When an article is published we post the peer reviewers' comments and the authors' responses online. We also post the versions of the paper that were used during peer review. These are the versions that the peer review comments apply to.

The versions of the paper that follow are the versions that were submitted during the peer review process. They are not the versions of record or the final published versions. They should not be cited or distributed as the published version of this manuscript.

BMJ Open is an open access journal and the full, final, typeset and author-corrected version of record of the manuscript is available on our site with no access controls, subscription charges or pay-per-view fees (http://bmjopen.bmj.com).

If you have any questions on BMJ Open's open peer review process please email info.bmjopen@bmj.com

BMJ Open

# Three common nutritional screening tools in the light of the new ESPEN consensus definition of the diagnostic criteria for malnutrition among geriatric gastrointestinal cancer patients



| | |
|---|---|
| Journal: | *BMJ Open* |
| Manuscript ID | bmjopen-2017-019750 |
| Article Type: | Research |
| Date Submitted by the Author: | 25-Sep-2017 |



| | |
|---|---|
| Complete List of Authors: | Ye, Xiao-Jun; Shanghai Tenth People's Hospital Affiliated to Tongji University, Department of Gastrointestinal Surgery; Medical College of Soochow University, Department of Medicine<br>Ji, Yan-Bin; Shanghai Tenth People's Hospital Affiliated to Tongji University, Department of Gastrointestinal Surgery; Shanghai Clinical Institution, Anhui Medical University, Department of Gastrointestinal Surgery<br>Ma, Bing-Wei; Shanghai Tenth People's Hospital Affiliated to Tongji University, Department of Gastrointestinal Surgery<br>Huang, Dong-Dong; Shanghai Tenth People's Hospital Affiliated to Tongji University, Department of Gastrointestinal Surgery<br>Chen, Wei-Zhe; The First Affiliated Hospital, Wenzhou Medical University, Department of Gastrointestinal Surgery<br>Pan, Zong-You; School of Medicine, Zhejiang University, Department of Sports Medicine<br>Shen, Xian; The Second Affiliated Hospital, Wenzhou Medical University, Department of Gastrointestinal Surgery<br>Zhuang, Cheng-Le; Shanghai Tenth People's Hospital Affiliated to Tongji University, Department of Gastrointestinal Surgery<br>Yu, Zhen; Shanghai Tenth People's Hospital Affiliated to Tongji University, Department of Gastrointestinal Surgery |



| | |
|---|---|
| <b>Primary Subject Heading</b>: | Gastroenterology and hepatology |
| Secondary Subject Heading: | Nutrition and metabolism, Oncology |
| Keywords: | Malnutrition, nutritional screening tools, ESPEN diagnostic criteria, gastrointestinal cancer, elderly |
| | |


SCHOLARONE™ Manuscripts

# Three common nutritional screening tools in the light of the new ESPEN consensus definition of the diagnostic criteria for malnutrition among geriatric gastrointestinal cancer patients

Xiao-Jun Ye, MD[1,5¶], Yan-Bin Ji, MD[1,6¶], Bing-Wei Ma, MD[1], Dong-Dong Huang, MD[1], Wei-Zhe Chen, MD[2], Zong-You Pan, MD[3], Xian Shen, MD, PhD[4], Cheng-Le Zhuang, MD, PhD[1*], and Zhen Yu, MD, PhD[1,2*]

[1] Department of Gastrointestinal Surgery, Shanghai Tenth People's Hospital Affiliated to Tongji University, Shanghai, China;

[2] Department of Gastrointestinal Surgery, The First Affiliated Hospital, Wenzhou Medical University, Wenzhou, Zhejiang, China;

[3] Department of Sports Medicine, School of Medicine, Zhejiang University, Hangzhou, Zhejiang, China;

[4] Department of Gastrointestinal Surgery, The Second Affiliated Hospital, Wenzhou Medical University, Wenzhou, Zhejiang, China;

[5] Medical College of Soochow University, Suzhou, Jiangsu, China;

[6] Department of Gastrointestinal Surgery, Shanghai Clinical Institution, Anhui Medical University, Shanghai, China.

¶These authors contributed equally to this work.

For peer review only

* Corresponding author:

E-mail: yuzhen0577@gmail.com (YZ); zhuangchengle@126.com (ZCL).

**Correspondence address:**

Department of Gastrointestinal Surgery, Shanghai Tenth People's Hospital affiliated to Tongji University, 301 Yanchang Road, Shanghai 200072, China

Tel.: +86 02166307132; fax: +86 02159410409.

**Funders:** This work was funded by the Science and Technology Commission of Shanghai Municipality [grant number 16411954200]; and Shanghai Municipal Commission of Health and Family Planning [grant number 201640013].

**All authors reported no biomedical financial interests or potential conflicts of interest.**

For peer review only

# Abstract

Objective: The aim of this study was to evaluate and compare the three common nutritional screening tools with the new ESPEN diagnostic criteria for malnutrition among the elderly gastrointestinal cancer patients.

Research Methods & Procedures: Nutritional screening tools, including Nutritional Risk Screening 2002 (NRS 2002), Malnutrition Universal Screening Tool (MUST) and the Short Form of Mini Nutrition Assessment (MNA-SF), were applied to 255 patients with gastrointestinal cancer. We compared the diagnostic value of these tools for malnutrition, using the new ESPEN diagnostic criteria for malnutrition as the "gold standards".

Results: According to the new ESPEN diagnostic criteria for malnutrition, 20.0% of the patients were diagnosed as malnourished. With the use of NRS 2002, 52.2% of the patients were found to be at high risk of malnutrition, with the use of MUST, 37.6% of the patients were found to be at moderate/high risk of malnutrition, and according to MNA-SF, 47.8% of the patients were found to be at nutritional risk. MUST was the best correlated with the ESPEN diagnostic criteria ($K = 0.530$, $p < 0.001$) compared with NRS 2002 ($K = 0.312$, $p < 0.001$) and MNA-SF ($K = 0.380$, $p < 0.001$). The ROC curve of MUST had the highest Area Under the Curve (AUC) compared with NRS 2002 and MNA-SF.

Conclusions: MUST was found to perform the best to identify the malnourished elderly gastrointestinal cancer patients distinguished by the new ESPEN diagnostic criteria for malnutrition. Nevertheless, further studies are needed to verify our findings.

Keywords: Malnutrition, nutritional screening tools, ESPEN diagnostic criteria, elderly, gastrointestinal cancer

## Strengths and limitations of this study

- To our best knowledge, this is the first study to evaluate the three screening tools in the specific geriatric gastrointestinal cancer patients.
- We compared the diagnostic value of the three screening tools, using the new ESPEN diagnostic criteria for malnutrition as the "gold standards".
- the sample size is relatively small. However, this study was conducted in two centers with large surgical volume. To a certain extent, it overcame the smallness of the sample size.

For peer review only

# Introduction

As the life expectancy and world population ages increase, the proportion of elderly patients has been enlarged obviously. It is well known that the risk of cancer increases with age. More than half of the malignancies occur in people aged ≥ 65 years.[1, 2] Gastrointestinal cancer is one of the most common malignancies in the elderly,[3] and surgical excision remains the most effective therapy for gastrointestinal cancers.[4] Although the surgical techniques have been improved significantly, elderly gastrointestinal cancer patients still have a high frequency of complications and mortality.[4-8] This is partly due to the high prevalence of malnutrition, which is a common and serious problem in the elderly cancer patients.[9, 10, 11] Therefore, it is important to evaluate the nutritional risk of the geriatric gastrointestinal cancer patients before surgery.

To accurately assess the nutritional risk, it is important to choose an efficient nutritional screening tool. Although there are many widely used nutritional screening tools,[12] such as Nutritional Risk Screening 2002 (NRS 2002),[13] Malnutrition Universal Screening Tool (MUST)[14] and the Short Form of Mini Nutritional Assessment (MNA-SF),[15] it has not been established which is the most efficient and appropriate for nutritional screening in the elderly patients with gastrointestinal cancers.

For peer review only

Moreover, there is a lack of universal definition of malnutrition, which may lead to an inaccurate assessment and comparison of the nutritional screening tools.

Recently, a diagnostic criteria for malnutrition has been proposed by the ESPEN,[16] and it had been validated by some studies.[17, 18] As the new criteria of defining malnutrition being proposed, it provides a reference standard for the evaluation and comparison of the nutritional screening tools. Therefore, our study was developed to evaluate the consistency of the three common nutritional screening tools with the new ESPEN diagnostic criteria for malnutrition and make a comparison of them among geriatric gastrointestinal cancer patients.

## Materials and Methods

### Patients

Between January 2016 and May 2017, 255 patients who underwent curative surgery for gastrointestinal cancer in two hospitals from Shanghai and Wenzhou were included in this study. The inclusion criteria included: (1) those underwent elective curative surgery for gastrointestinal cancer; (2) those aged $\geq$ 70 years; (3) those signed the informed consent and agreed to participate in this study. The exclusion criteria included: (1) those performed a palliative or emergency operation; (2) those aged < 70 years; (3) those cannot be assessed by NRS 2002,

For peer review only

MUST and MNA-SF for the difficulty of data collection; (4) those refused to take part in this study.

This study was approved by the ethics committees of the Tenth Affiliated Hospital of Tongji University and the First Affiliated Hospital of Wenzhou Medical University.

## Data collection

The general and anthropometric data of the patients were collected. The general data contained the parameters of age, sex, diagnosis, morbidity, the change of appetite and physical activity. The anthropometric parameters included weight, height, unintentional weight loss, and body mass index (BMI).

## Reference standard: the new ESPEN diagnostic criteria for malnutrition

According to the new ESPEN diagnostic criteria [16], malnutrition was diagnosed when the patients met one of the following two options. Option one required BMI < 18.5 kg/$m^2$. Option two required unintentional weight loss > 10% indefinite of time or > 5% over the last three months combined with reduced BMI (< 20 kg/$m^2$ in patients younger than 70 years or < 22 kg/$m^2$ in patients older than 70 years).

## Assessment of nutritional risk

The assessment was performed by using the following nutritional screening tools: NRS 2002, MUST, and MNA-SF.

For peer review only

NRS 2002 is proposed by the ESPEN guidelines based on an analysis of controlled clinical trials [13]. It is designed to identify who would benefit from nutritional supporting. This tool contains a severity of disease score, a nutritional score and an age score. Severity of disease score: one point for hip fracture, long-term hemodialysis, diabetes mellitus, or chronic disease with acute complications; two points for major abdominal surgery, hematological malignancies, stroke, or severe pneumonia; three points for head injury, bone marrow transplantation, or intensive care patients with APACHE more than 10. Nutritional score: one point for weight loss > 5% in 3 months or food intake 50-75% of the common condition; two points for weight loss > 5% in 2 months or BMI 18.5-20.5 kg/m$^2$ with impaired general condition or food intake 25-60% of the general condition; three points for weight loss > 5% in 1 month or BMI less than 18.5 kg/m$^2$ with impaired general condition or food intake reduced by 25% compared with normal condition. Age score: one point for age ≥ 70 years. Nutritional risk was assessed by summarizing the severity disease score, nutritional score and the age score. Patients with total score < 3 are at no or low risk, and with score ≥ 3 are at high risk.

MUST is developed to assess the nutritional risk for adults [14]. It consists of parameters of BMI, unintentional weight loss and any acute disease which compromises nutritional intake for more than 5 days. The three parameters are rated as 0, 1, or 2 as follows: BMI > 20 kg/m$^2$ = 0;

For peer review only

18.5-20 kg/m$^2$ = 1; < 18.5 kg/m$^2$ = 2; unintentional weight loss in the past 3-6 months < 5% = 0; 5-10% = 1; > 10% = 2; acute disease: absent = 0; present = 2. Overall risk of malnutrition is assessed by adding all the points together. 0 is at low risk; score 1 is at medium risk; and score 2 is at high risk.

MNA-SF is the short form of MNA, and it is designed especially for the elderly. It contains six questions selected from MNA.[15] These questions are about BMI, recent weight loss, change of appetite, mobility, psychological stress and neuropsychological problems. Each question is rated from 0 to 2 or 3 and the total score of MNA-SF is 14. Patients with 12-14 points are at normal nutritional status. And patients with scores ≤ 11 are at risk of malnutrition.

## Statistical analysis

Statistical analysis was performed using SPSS software version 23.0 for windows. Normally distributed continuous variables were expressed as mean values and standard deviations (SD), categorical variables were presented as absolute and relative frequencies. Independent t test and the Pearson $\chi^2$ test (or Fisher's exact test) were applied to the appropriate comparison of variables. All reported P-values were compared to a significance level of 5% based on two-sided tests. To determine diagnostic concordance between the three assessment tools and the new ESPEN diagnostic criteria for malnutrition, Cohen's kappa (κ) statistic

For peer review only

was calculated. Κ coefficient reflects the consistency for qualitative variables. Κ = 1 means completely consistency between the variables. And if there is no consistency among the variables then Κ ≤ 0. Positive likelihood ratios (LR+) and negative likelihood ratios (LR-) were calculated for all three tools.

Sensitivity and specificity values for the three nutritional screening tools with the new ESPEN diagnostic criteria for malnutrition were calculated. Receiver operating characteristic curves (ROC) of the three screening tools were also used to evaluate the ability to accurately distinguish the malnourished patients. Area under the ROC curer (AUC) equal to 0.5 indicates that a tool has no diagnostic value, AUC equal to 0.5-0.7 indicates a tool has a low diagnostic value, AUC equal to 0.7-0.9 indicates a tool has a moderate diagnostic value, and AUC equal to 0.9-1 means a tool has a high diagnostic value.

## Results

Two hundred and fifty five patients were enrolled in this study (103 patients underwent gastric cancer surgery and 152 patients underwent colorectal cancer surgery). The characteristics of the sample were presented in Table 1.

**Table 1. Characteristics of the total patients.**

| Patient Characteristics | Total (n=255) | GC (n=103) | CRC (n=152) | P value |
|---|---|---|---|---|
| Age [a] (years) | 76.5±4.8 | 76.1±4.6 | 76.8±4.9 | 0.318 |
| Sex [b] | | | | |
| Male | 160(62.7%) | 78(75.7%) | 82(52.1%) | <0.001* |
| Female | 95(37.3%) | 25(24.3%) | 70(47.9%) | |

For peer review only

| | | | | |
|---|---|---|---|---|
| Height [a] (m) | 1.61±0.08 | 1.61±0.07 | 1.60±0.09 | 0.269 |
| Weight [a] (kg) | 59.20±10.73 | 59.67±10.26 | 58.90±11.07 | 0.597 |
| BMI [a] (kg/$m^2$) | 22.93±3.55 | 22.93±3.50 | 22.96±3.59 | 0.994 |
| BMI [b] (＜18.5kg/$m^2$) | 26(10.2%) | 11(10.7%) | 15(9.9%) | 0.834 |
| Weight loss [b] (＞5% in 3 months or ＞10% indefinite of time) | 74(29.0%) | 31(30.1%) | 43(28.3%) | 0.755 |

BMI: body mass index. GC: gastric cancer. CRC: colorectal cancer. *Statistically significance ($p \leq 0.05$).

[a] Values expressed as mean ± standard deviation(SD) .

[b] Values expressed as frequencies and percentages.

P values were determined with the use of independent t test and the Pearson$\chi^2$ test.

Table 2 listed the characteristics and anthropometric data of the patients summarized and stratified according to the nutritional status. There were no difference in age or sex between the two groups classified by the three screening tools and the new ESPEN criteria for malnutrition. However, the BMI and weight loss (> 5% in 3 months or > 10% indefinite of time) differed between the groups.

For peer review only

Table 2. Characteristics and anthropometric data stratified by the nutrition status of total patients.

| | ESPEN criteria | | | NRS 2002 | | | MUST | | | MNA-SF | | |
|---|---|---|---|---|---|---|---|---|---|---|---|---|
| | Not malnourished (n=204) | Malnourished (n=51) | P value | No or Low risk (n=122) | High risk (n=133) | P value | Low risk (n=159) | Moderate/High risk (n=96) | P value | No risk (n=133) | Risk of malnutrition (n=122) | P value |
| Age [a] (years) | 76.3±4.8 | 77.4±4.9 | 0.148 | 75.9±4.5 | 77.0±5.0 | 0.059 | 76.1±4.6 | 77.1±5.1 | 0.097 | 76.0±4.6 | 77.0±5.0 | 0.102 |
| Sex [b] | | | | | | | | | | | | |
| male | 124(60.8%) | 36(70.6%) | 0.195 | 73(59.8%) | 87(65.4%) | 0.357 | 101(63.5%) | 59(61.5%) | 0.741 | 90(67.7%) | 70(57.4%) | 0.089 |
| female | 80(39.2%) | 15(29.4%) | | 49(40.2%) | 46(34.6%) | | 58(36.5%) | 37(38.5%) | | 43(32.3%) | 52(42.6%) | |
| Height [a] (m) | 1.60±0.08 | 1.63±0.08 | 0.046* | 1.60±0.08 | 1.61±0.09 | 0.155 | 1.60±0.08 | 1.61±0.09 | 0.511 | 1.61±0.08 | 1.60±0.08 | 0.112 |
| Weight [a] (kg) | 61.55±10.00 | 49.80±8.17 | <0.001* | 60.23±10.04 | 58.26±11.29 | 0.142 | 62.46±9.55 | 53.80±10.45 | <0.001* | 63.32±9.56 | 54.72±10.16 | <0.001* |
| BMI [a] (kg/m$^2$) | 23.98±3.01 | 18.76±2.20 | <0.001* | 23.56±3.27 | 22.36±3.70 | 0.006* | 24.28±2.95 | 20.71±3.34 | <0.001* | 24.30±2.98 | 21.45±3.52 | <0.001* |
| BMI [b] (<18.5kg/m$^2$) | 1(0.5%) | 25(49.0%) | <0.001* | 4(3.3%) | 22(16.5%) | 0.001* | 1(0.6%) | 25(26.0%) | <0.001* | 1(0.8%) | 25(20.5%) | <0.001* |
| Weight loss [b] (>5% in 3 months or >10% indefinite of time) | 34(16.7%) | 40(78.4%) | <0.001* | 4(3.3%) | 70(52.6%) | <0.001* | 14(8.8%) | 60(62.5%) | <0.001* | 4(3.0%) | 70(57.4%) | <0.001* |

*Statistically significance ($p \leq 0.05$).

[a] Values expressed as mean ± standard deviation(SD) .

[b] Values expressed as frequencies and percentages.

P values were determined with the use of independent t test and the Pearson $\chi^2$ test(or Fisher's exact test where appropriate). BMI: body mass index.

The classification of malnutrition according to the new ESPEN criteria and the classification of nutritional risk according to the three screening tools were shown in Table 3. Among the patients, the prevalence of malnutrition was 20.0% when determined by the ESPEN criteria. The rate of high risk of malnutrition was 52.2% when determined by NRS 2002,



For peer review only

37.6% when determined by MUST, and 47.8% when determined by MNA-SF. Among patients who underwent curative gastrectomy, the prevalence of malnutrition was 22.3% when determined by the new ESPEN criteria. The rates of moderate or high risk of malnutrition were 52.4%, 45.6%, and 43.7% with NRS 2002, MUST, and MNA-SF respectively. Among colorectal surgery patients, the prevalence of malnutrition was 18.4% when determined by the new ESPEN criteria, the rates of moderate and high risk of malnutrition were 52.0%, 32.2%, and 50.7% with NRS 2002, MUST and MNA-SF, respectively.

**Table 3. Classification of the risk of malnutrition with the ESPEN criteria and the three screening tools.**

| Risk of malnutrition | Total | | | | GC (n=103) | | | | CRC (n=152) | | | |
|---|---|---|---|---|---|---|---|---|---|---|---|---|
| | ESPEN criteria [a] | NRS 2002 | MUST | MNA-SF | ESPEN criteria [a] | NRS 2002 | MUST | MNA-SF | ESPEN criteria [a] | NRS 2002 | MUST | MNA-SF |
| No/Low | 80.0% (204/255) | 47.8% (122/255) | 62.4% (159/255) | 52.2% (133/255) | 77.7% (80/103) | 47.6% (49/103) | 54.4% (56/103) | 56.3% (58/103) | 81.6% (124/152) | 48.0% (73/152) | 67.8% (103/152) | 49.3% (75/152) |
| Moderate/High | 20.0% (51/255) | 52.2% (133/255) | 37.6% (96/255) | 47.8% (122/255) | 22.3% (23/103) | 52.4% (54/103) | 45.6% (47/103) | 43.7% (45/103) | 18.4% (28/152) | 52.0% (79/152) | 32.2% (49/152) | 50.7% (77/152) |

GC: gastric cancer. CRC: colorectal cancer.

a Classification of malnutrition according to the ESPEN consensus definition of malnutrition.

Cross tabulation of the results of the three tools and the classification of malnutrition according to the ESPEN

For peer review only

consensus definition of malnutrition can be found in Table 4.

**Table 4. Cross tabulation of the results of the three screening tools and the classification of malnutrition according to the ESPEN consensus definition of malnutrition.**

| | | NRS 2002 | | MUST | | MNA-SF | |
|---|---|---|---|---|---|---|---|
| | | No/Low risk | Moderate/High risk | Low risk | Moderate/High risk | No risk | Risk |
| ESPEN criteria | Not Malnourished | 118 | 86 | 156 | 48 | 130 | 74 |
| | Malnourished | 4 | 47 | 3 | 48 | 3 | 48 |

The consistency between the nutritional screening tools and the new ESPEN diagnostic criteria for malnutrition was different. Among the total patients, MUST and MNA had the same sensitivity (94.1%), and NRS 2002 had the lowest sensitivity (92.2%). Moreover, MUST had the highest specificity (76.5%) compared with NRS 2002 (57.8%) and MNA-SF (63.7%). MUST had the highest positive predictive value (50.0%) and the highest negative predictive value (98.1%).

In the total patients, MUST had the highest К value (К = 0.530, $p<0.001$) compared with MNA-SF (К = 0.380, $p<0.001$) and NRS 2002 (К = 0.312, $p<0.001$). In the gastric group, MUST had the highest К value. In the colorectal group, MUST had a higher level of consistency (К = 0.576, $p<0.001$) compared with the fair consistencies in NRS 2002 (К = 0.243, $p<0.001$) and MNA-SF (К = 0.361, $p<0.001$). Finally, the area under the curve (AUC) calculated by the ROC

For peer review only

indicated that all three screening tools had a moderate level of diagnostic value to distinguish a malnourished patient (AUC of NRS 2002, MUST and MNA-SF were found to be 0.750, 0.853 and 0.789, respectively). Results are presented in details in Table 5.

**Table 5. Statistical evaluation of the malnutrition screening tools compared with the diagnostic criteria of the ESPEN consensus.**

| | Total | | | GC | | | CRC | | |
|---|---|---|---|---|---|---|---|---|---|
| | NRS 2002 | MUST | MNA-SF | NRS 2002 | MUST | MNA-SF | NRS 2002 | MUST | MNA-SF |
| Sensitivity (%) | 92.2 | 94.1 | 94.1 | 100.0 | 95.7 | 87.0 | 85.7 | 92.9 | 100.0 |
| Specificity (%) | 57.8 | 76.5 | 63.7 | 61.3 | 68.8 | 68.8 | 55.6 | 81.5 | 60.5 |
| Positive predictive value (%) | 35.3 | 50.0 | 39.3 | 42.6 | 46.8 | 44.4 | 30.4 | 53.1 | 36.4 |
| Negative predictive value (%) | 96.7 | 98.1 | 97.7 | 100.0 | 98.2 | 94.8 | 94.5 | 98.1 | 100.0 |
| Positive likelihood ratio (LR+) | 2.18 | 4.00 | 2.59 | 2.61 | 3.06 | 2.78 | 1.93 | 5.02 | 2.53 |
| Negative likelihood ratio (LR-) | 0.13 | 0.08 | 0.09 | 0.00 | 0.06 | 0.19 | 0.26 | 0.09 | 0.00 |
| K value (p) | 0.312(＜0.001) | 0.530(＜0.001) | 0.380(＜0.001) | 0.414(＜0.001) | 0.469(＜0.001) | 0.415(＜0.001) | 0.243(＜0.001) | 0.576(＜0.001) | 0.361(＜0.001) |
| AUC | 0.750 | 0.853 | 0.789 | 0.806 | 0.822 | 0.779 | 0.707 | 0.872 | 0.802 |

GC: gastric cancer. CRC: colorectal cancer.

K value derived from Cohen kappa statistics.

AUC: Area under the curve from ROC.

For peer review only

# Discussion

This study is the first to apply the new ESPEN diagnostic criteria to the specific population of elderly gastrointestinal cancer patients, and 20.0% of the patients were classified as malnourished according to the new ESPEN diagnostic criteria for malnutrition. Another previous study has investigated the prevalence of malnutrition diagnosed by the new ESPEN criteria in four diverse populations.[19] In that study, 0.5% of the healthy elderly individuals and 6% of the geriatric outpatients were identified as malnourished. It is significantly lower compared with that of our study, which indicated that patients with gastrointestinal cancer might have a higher prevalence of malnutrition. The difference in the malnutrition rates also indicated that we should put emphasis on the requirement to assess the nutritional risk of the hospitalized elderly gastrointestinal cancer patients.

In the present study, 52.2% and 37.6% of the patients were found to be at moderate or high risk of malnutrition according to NRS 2002 and MUST respectively. With the MNA-SF, 47.8% of the patients were found to be at a risk of malnutrition. The various prevalence of the risk of malnutrition can result from the differences between the nutritional screening tools. In our study, MUST had the greatest K value compared with the NRS 2002 and MNA-SF. It showed that a greater proportion of the elderly patients with gastrointestinal cancer who were distinguished to

For peer review only - http://bmjopen.bmj.com/site/about/guidelines.xhtml

be at moderate or high risk of malnutrition with MUST could be identified as malnourished according to the ESPEN diagnostic criteria. In other words, MUST can perform the best to detect the specific malnourished individuals diagnosed by the new ESPEN criteria, compared with NRS 2002 and MNA-SF. Furthermore, MUST has the greatest AUC compared with NRS 2002 and MNA-SF in our study.

Many previous studies compared the three nutritional screening tools in specific populations. Poulia et al. evaluated the efficacy of six nutritional screening tools in the elderly.[20] In Poulia' s study, NRS 2002 was found to overestimate nutritional risk, MNA-SF was proven to have a great validity, and MUST was found to have the best validity and the greatest consistency. Another study by Myoungha et al.[21] evaluated five nutritional screening tools, and suggested that MNA-SF overestimated the nutritional risk in the elderly, and NRS 2002 performed better than MNA-SF. However, MUST was also found to be the most efficient and useful screening tool in this study. Both the previous studies compared the screening tools with a combined index suggested by Pablo et al.,[22] and they confirmed our results for the best performance of MUST compared with NRS 2002 and MNA-SF. While Donini et al. developed a study for the nutritional evaluation of the elderly nursing home residents[23] and found that MNA-SF presented a higher predictive value compared with NRS 2002 and MUST. However, in the study by Donini et

For peer review only

al., MNA was taken as the reference standard, which might induce an underpowered result. A previous study used the new ESPEN diagnostic criteria for malnutrition as a reference standard to compare nutritional screening tools.[24] However, MNA-SF was not included in this study, and the participators were not merely the elderly.

The results of the comparison among NRS 2002, MUST and MNA-SF showed different efficiency of the three screening tools in our study. There might be an explanation for the differences. The original designs of the three nutritional screening tools were different. NRS 2002 was developed to determine who would benefit from nutritional supporting and it might distinguish an increased number of patients to be at high risk of malnutrition. However, MUST was a screening tool to identify adults who are at risk of malnutrition. MNA-SF, the short form of MNA, was developed as a quick and easy nutritional screening tool and was used for primary screening before further assessment. The different results between our study and others can be attributed to the different populations and reference standards, just as what Ma et al. had mentioned in their review.[25]

Moreover, in our study, we evaluated the adequacy of the nutritional screening tools in the gastric cancer patients and the colorectal cancer patients, respectively. In the gastric cancer population, NRS 2002, MUST and MNA-SF all had a same moderate level of consistency with the new

For peer review only

ESPEN diagnostic criteria for malnutrition (К = 0.414 for NRS 2002, 0.469 for MUST and 0.415 for MNA-SF, respectively). While in the colorectal population, MUST had the highest level of consistency (К = 0.576) compared with the fair level of consistency of NRS 2002 (К = 0.243) and MNA-SF (К = 0.361). Based on the result, we concluded that MUST could perform the best in the specific colorectal cancer patients. Further studies are needed to confirm our conclusion.

It is of great significance to improve the nutritional status of the elderly patients with gastrointestinal cancer if they are malnourished or at risk of malnutrition. As the first step to identify who are malnourished or at risk of malnutrition, nutritional screening should be reliable and easy to perform. To the best of our knowledge, this is the first study to compare the three nutritional screening tools in the specific geriatric gastrointestinal cancer population. Our study suggested that MUST is the best choice in the three common nutritional screening tools. For the accuracy and simplicity of MUST, both the patients and surgeons will benefit from it. Furthermore, in our study, though there were some differences in sensitivity and specificity, the three tools were found to have a same level of consistency in the gastric cancer population, while in the colorectal cancer population, MUST had the highest level of consistency compared with the others. It indicated that we can choose one of three nutritional screening tools when assessing nutritional risk among

For peer review only

the elderly patients with gastric cancer. However, when assessing nutritional risk among the elderly patients with colorectal cancer, MUST is the only choice for the highest accuracy.

The present study has some limitations. Firstly, the sample size is relatively small. However, this study was conducted in two centers with large surgical volume. So we believe that the data in our study are more representative. To a certain extent, it overcame the smallness of the sample size. Secondly, according to the new ESPEN diagnostic criteria for malnutrition, malnutrition can also be diagnosed by unintentional weight loss combined with reduced fat free mass index (FFMI). So another limitation of our study is the lack of data for fat free mass index. However, the measurement of FFMI requires specific equipment and extra costs. Moreover, Trummer et al. found that low BMI and low FFMI were closely correlative.[25] In that study, FFMI less than 17 $kg/m^2$ for men and less than 15 $kg/m^2$ for women were roughly equivalent to BMI less than 18.5 $kg/m^2$ after determining the FFM levels.

## Conclusions

To our knowledge, this is the first study to compare the three malnutrition screening tools (NRS 2002, MUST and MNA-SF) with the new ESPEN diagnostic criteria for malnutrition, and it is also the first study to evaluate the three screening tools in the specific geriatric gastrointestinal cancer patients. The prevalence of malnutrition was 20.0%

For peer review only

with the ESPEN diagnostic criteria for malnutrition for patients with gastrointestinal cancer in the present study. MUST was found to perform the best to identify the malnourished elderly gastrointestinal cancer patients distinguished by the new ESPEN diagnostic criteria for malnutrition. Nevertheless, further studies are needed to verify our findings.

## References


1. Hurria A, Gupta S, Zauderer M, et al. Developing a cancer-specific geriatric assessment: a feasibility study. Cancer 2005;104:1998-2005. doi:10.1002/cncr.21422.
2. Balducci L, Extermann M. Cancer and aging. An evolving panorama. Hematology/oncology Clinics of North America 2000;14:1-16.
3. Marugame T, Dongmei Q. Comparison of Time Trends in Stomach Cancer Incidence (1973–1997) in East Asia, Europe and USA, from Cancer Incidence in Five Continents Vol. IV–VIII. Jpn J Clin Oncol. 2007;37:242-3. doi:10.1093/jjco/hym014.
4. Biondi A, Vacante M, Ambrosino I, et al. Role of surgery for colorectal cancer in the elderly. World Journal of Gastrointestinal Surgery 2011;8:606-13. doi:10.4240/wjgs.v8.i9.606
5. Takeshita H, Ichikawa D, Komatsu S, et al. Surgical outcomes of gastrectomy for elderly patients with gastric cancer. World Journal of


For peer review only

Surgery 2013;37:2891-8. doi: 10.1007/s00268-013-2210-7.

6. Simmonds PD, Best L, George S, et al. Surgery for colorectal cancer in elderly patients: a systematic review. Lancet 2000;356:968-74. doi:http://dx.doi.org/10.1016/S0140-6736(00)02713-6.
7. Heriot AG, Tekkis PP, Smith JJ, et al. Prediction of postoperative mortality in elderly patients with colorectal cancer. Diseases of the Colon & Rectum 2006;49:816-24. doi: 10.1007/s10350-006-0523-4.
8. Audisio RA, Balch CM. Why Can't Surgeons Treat Older Patients the Same as Younger Patients? Annals of Surgical Oncology 2016;23:4123-5. doi:10.1245/s10434-016-5459-x.
9. Brownie S. Why are elderly individuals at risk of nutritional deficiency? International Journal of Nursing Practice 2006, 12:110-8. doi:10.1111/j.1440-172X.2006.00557.x.
10. Bozzetti F. Why the oncologist should consider the nutritional status of the elderly cancer patient. Nutrition 2015;31:590-3. doi:10.1016/j.nut.2014.12.005.
11. Martucci RB, Barbosa MV, D'Almeida CA, et al. Undernutrition as independent predictor of early mortality in elderly cancer patients. Nutrition 2017;34:65-70. doi:10.1016/j.nut.2016.09.011.
12. Kondrup J, Allison SP, Elia M, et al. ESPEN guidelines for nutrition screening 2002. Clinical Nutrition 2003;22:415-21. doi:10.1016/S0261-5614(03)00098-0.

13. Hamberg O. Nutritional risk screening (NRS 2002): a new method based on an analysis of controlled clinical trials. Clinical Nutrition 2003;22:321-36. doi:10.1016/S0261-5614(02)00214-5.

14. Elia M. The 'MUST' report. Nutritional screening for adults: a multidisciplinary responsibility. Development and use of the 'Malnutrition Universal Screening Tool' (MUST) for adults. British Association for Parenteral and Enteral Nutrition (BAPEN) 2003.

15. Rubenstein LZ, Harker JO, Salvà A, et al. Screening for undernutrition in geriatric practice: developing the short-form mini-nutritional assessment (MNA-SF). Journals of Gerontology 2001;56:M366-72.

16. Cederholm T, Bosaeus I, Barazzoni R, et al. Diagnostic criteria for malnutrition - An ESPEN Consensus Statement. Clinical Nutrition 2015;139:335-40. doi:10.1016/j.clnu.2015.03.001.

17. Guerra RS, Fonseca I, Sousa AS, et al. ESPEN diagnostic criteria for malnutrition – A validation study in hospitalized patients. Clinical Nutrition 2016 [Epub ahead of print] doi:10.1016/j.clnu.2016.08.022.

18. Rondel AL, Langius JA, de van der Schueren MA, et al. The new ESPEN diagnostic criteria for malnutrition predict overall survival in hospitalised patients. Clinical Nutrition 2016 [Epub ahead of print] doi:10.1016/j.clnu.2016.11.018.

19. Rojer AG, Kruizenga HM, Trappenburg MC, et al. The prevalence of

For peer review only

malnutrition according to the new ESPEN definition in four diverse populations. Clinical Nutrition 2015;35:758-62. doi:10.1016/j.clnu.2015.06.005.

20. Poulia KA, Yannakoulia M, Karageorgou D, et al. Evaluation of the efficacy of six nutritional screening tools to predict malnutrition in the elderly. Clinical Nutrition 2012;31:378-85. doi:10.1016/j.clnu.2011.11.017.
21. Myoungha B, Youngran H. Evaluation of the efficacy of nutritional screening tools to predict malnutrition in the elderly at a geriatric care hospital. Nutrition Research & Practice 2015;9:637-43. doi:10.4162/nrp.2015.9.6.637.
22. Pablo AM, Izaga MA, Alday LA. Assessment of nutritional status on hospital admission: nutritional scores. European Journal of Clinical Nutrition 2003;57:824-31. doi:10.1038/sj.ejcn.1601616.
23. Donini LM, Poggiogalle E, Molfino A, et al. Mini-Nutritional Assessment, Malnutrition Universal Screening Tool, and Nutrition Risk Screening Tool for the Nutritional Evaluation of Older Nursing Home Residents. J Am Med Dir Assoc 2016;17:959.e11-8. doi:10.1016/j.jamda.2016.06.028.
24. Poulia KA, Klek S, Doundoulakis I, et al. The two most popular malnutrition screening tools in the light of the new ESPEN consensus definition of the diagnostic criteria for malnutrition. Clinical Nutrition

For peer review only

2017;36:1130-5. doi:10.1016/j.clnu.2016.07.014.

25. van Bokhorst-de van der Schueren MA, Guaitoli PR, Jansma EP, et al. Nutrition screening tools: does one size fit all? A systematic review of screening tools for the hospital setting. Clinical Nutrition 2014;33:39-58. doi:10.1016/j.clnu.2013.04.008.

26. Bahadori B, Uitz E, Tonninger-Bahadori K, et al. Body composition: the fat-free mass index (FFMI) and the body fat mass index (BFMI) distribution among the adult Austrian population-results of a cross-sectional pilot study. International Journal of Body Composition Research 2006;4:123-8.

**Contributors:** Xiao-Jun Ye and Yan-Bin Ji are joint first authors. Zhen Yu, Cheng-Le Zhuang, and Xian Shen designed the study. Bing-Wei Ma and Wei-Zhe Chen collected the data. Dong-Dong Huang and Zong-You Pan analyzed the data. Xiao-Jun Ye and Yan-Bin Ji drafted the manuscript.Zhen Yu and Cheng-Le Zhuang approved the final version of the manuscript. All authors have read and approved the final manuscript.

**Data sharing statement:** No additional unpublished data are available.

For peer review only

# Comparison of three common nutritional screening tools with the new European Society for Clinical Nutrition and Metabolism (ESPEN) criteria for malnutrition among geriatric gastrointestinal cancer patients: a prospective study in China

| | |
|---|---|
| Journal: | *BMJ Open* |
| Manuscript ID | bmjopen-2017-019750.R1 |
| Article Type: | Research |
| Date Submitted by the Author: | 05-Feb-2018 |
| Complete List of Authors: | Ye, Xiao-Jun; Shanghai Tenth People's Hospital Affiliated to Tongji University, Department of Gastrointestinal Surgery; Medical College of Soochow University, Department of Medicine<br>Ji, Yan-Bin; Shanghai Tenth People's Hospital Affiliated to Tongji University, Department of Gastrointestinal Surgery; Shanghai Clinical Institution, Anhui Medical University, Department of Gastrointestinal Surgery<br>Ma, Bing-Wei; Shanghai Tenth People's Hospital Affiliated to Tongji University, Department of Gastrointestinal Surgery<br>Huang, Dong-Dong; Shanghai Tenth People's Hospital Affiliated to Tongji University, Department of Gastrointestinal Surgery<br>Chen, Wei-Zhe; The First Affiliated Hospital, Wenzhou Medical University, Department of Gastrointestinal Surgery<br>Pan, Zong-You; School of Medicine, Zhejiang University, Department of Sports Medicine<br>Shen, Xian; The Second Affiliated Hospital, Wenzhou Medical University, Department of Gastrointestinal Surgery<br>Zhuang, Cheng-Le; Shanghai Tenth People's Hospital Affiliated to Tongji University, Department of Gastrointestinal Surgery<br>Yu, Zhen; Shanghai Tenth People's Hospital Affiliated to Tongji University, Department of Gastrointestinal Surgery |
| <b>Primary Subject Heading</b>: | Nutrition and metabolism |
| Secondary Subject Heading: | Gastroenterology and hepatology |
| Keywords: | Malnutrition, nutritional screening tools, ESPEN diagnostic criteria, elderly, gastrointestinal cancer |
| | |

SCHOLARONE™
Manuscripts

# Comparison of three common nutritional screening tools with the new European Society for Clinical Nutrition and Metabolism (ESPEN) criteria for malnutrition among geriatric gastrointestinal cancer patients: a prospective study in China

Xiao-Jun Ye, MD[1,5¶], Yan-Bin Ji, MD[1,6¶], Bing-Wei Ma, MD[1], Dong-Dong Huang, MD[1], Wei-Zhe Chen, MD[2], Zong-You Pan, MD[3], Xian Shen, MD, PhD[4], Cheng-Le Zhuang, MD, PhD[1], and Zhen Yu, MD, PhD[1,2]

[1] Department of Gastrointestinal Surgery, Shanghai Tenth People's Hospital Affiliated to Tongji University, Shanghai, China;

[2] Department of Gastrointestinal Surgery, The First Affiliated Hospital, Wenzhou Medical University, Wenzhou, Zhejiang, China;

[3] Department of Sports Medicine, School of Medicine, Zhejiang University, Hangzhou, Zhejiang, China;

[4] Department of Gastrointestinal Surgery, The Second Affiliated Hospital, Wenzhou Medical University, Wenzhou, Zhejiang, China;

[5] Medical College of Soochow University, Suzhou, Jiangsu, China;

[6] Department of Gastrointestinal Surgery, Shanghai Clinical Institution, Anhui Medical University, Shanghai, China.

¶These authors contributed equally to this work.



For peer review only - http://bmjopen.bmj.com/site/about/guidelines.xhtml

Correspondence to:

Dr Zhen Yu; yuzhen0577@gmail.com and Dr Cheng-Le Zhuang; zhuangchengle@126.com

**Correspondence address:**

Department of Gastrointestinal Surgery, Shanghai Tenth People's Hospital affiliated to Tongji University, 301 Yanchang Road, Shanghai 200072, China

Tel.: +86 02166307132; fax: +86 02159410409.

**Funders:** This work was funded by the Science and Technology Commission of Shanghai Municipality [grant number 16411954200]; and Shanghai Municipal Commission of Health and Family Planning [grant number 201640013].

**Registration Number: ChiCTR-RRC-16009831**

**All authors reported no biomedical financial interests or potential conflicts of interest.**

For peer review only



For peer review only - http://bmjopen.bmj.com/site/about/guidelines.xhtml

# Abstract

Objective: The aim of this study was to evaluate and compare the three common nutritional screening tools with the new European Society for Clinical Nutrition and Metabolism (ESPEN) diagnostic criteria for malnutrition among the elderly gastrointestinal cancer patients.

Research Methods & Procedures: Nutritional screening tools, including Nutritional Risk Screening 2002 (NRS 2002), Malnutrition Universal Screening Tool (MUST) and the Short Form of Mini Nutrition Assessment (MNA-SF), were applied to 255 patients with gastrointestinal cancer. We compared the diagnostic value of these tools for malnutrition, using the new ESPEN diagnostic criteria for malnutrition as the "gold standards".

Results: According to the new ESPEN diagnostic criteria for malnutrition, 20.0% of the patients were diagnosed as malnourished. With the use of NRS 2002, 52.2% of the patients were found to be at high risk of malnutrition, with the use of MUST, 37.6% of the patients were found to be at moderate/high risk of malnutrition, and according to MNA-SF, 47.8% of the patients were found to be at nutritional risk. MUST was the best correlated with the ESPEN diagnostic criteria ($K = 0.530$, $p < 0.001$) compared with NRS 2002 ($K = 0.312$, $p < 0.001$) and MNA-SF ($K = 0.380$, $p < 0.001$). The ROC curve of MUST had the highest Area Under the Curve (AUC) compared with NRS 2002 and MNA-SF.




For peer review only - http://bmjopen.bmj.com/site/about/guidelines.xhtml

Conclusions: MUST was found to perform the best to identify the malnourished elderly gastrointestinal cancer patients distinguished by the new ESPEN diagnostic criteria for malnutrition. Nevertheless, further studies are needed to verify our findings.

Keywords: Malnutrition, nutritional screening tools, ESPEN diagnostic criteria, elderly, gastrointestinal cancer

## Strengths and limitations of this study

- To our best knowledge, this is the first study to evaluate the three screening tools in the specific geriatric gastrointestinal cancer patients.
- We compared the diagnostic value of the three screening tools, using the new ESPEN diagnostic criteria for malnutrition as the "gold standards".
- the sample size is relatively small. However, this study was conducted in two centers with large surgical volume. To a certain extent, it overcame the smallness of the sample size.



For peer review only - http://bmjopen.bmj.com/site/about/guidelines.xhtml

# Introduction

As the life expectancy and world population ages increase, the proportion of elderly patients has been enlarged obviously. It is well known that the risk of cancer increases with age. More than half of the malignancies occur in people aged ≥ 65 years.[1, 2] Gastrointestinal cancer is one of the most common malignancies in the elderly,[3] and surgical excision remains the most effective therapy for gastrointestinal cancers.[4] Although the surgical techniques have been improved significantly, elderly gastrointestinal cancer patients still have a high frequency of complications and mortality.[4-8] This is partly due to the high prevalence of malnutrition, which is a common and serious problem in the elderly cancer patients.[9, 10, 11] Therefore, it is important to evaluate the nutritional risk of the geriatric gastrointestinal cancer patients before surgery.

To accurately assess the nutritional risk, it is important to choose an efficient nutritional screening tool. Although there are many widely used nutritional screening tools,[12] such as Nutritional Risk Screening 2002 (NRS 2002),[13] Malnutrition Universal Screening Tool (MUST)[14] and the Short Form of Mini Nutritional Assessment (MNA-SF),[15] it has not been established which is the most efficient and appropriate for nutritional screening in the elderly patients with gastrointestinal cancers.



For peer review only - http://bmjopen.bmj.com/site/about/guidelines.xhtml

Moreover, there is a lack of universal definition of malnutrition, which may lead to an inaccurate assessment and comparison of the nutritional screening tools.

Recently, a diagnostic criteria for malnutrition has been proposed by the European Society for Clinical Nutrition and Metabolism (ESPEN),[16] and it had been validated by some studies.[17, 18] As the new criteria of defining malnutrition being proposed, it provides a reference standard for the evaluation and comparison of the nutritional screening tools. Therefore, our study was developed to evaluate the consistency of the three common nutritional screening tools with the new ESPEN diagnostic criteria for malnutrition and make a comparison of them among geriatric gastrointestinal cancer patients.

# Materials and Methods

## Patients

Between January 2016 and May 2017, 255 patients who underwent curative surgery for gastrointestinal cancer in two hospitals from Shanghai and Wenzhou were included in this study. The inclusion criteria included: (1) those underwent elective curative surgery for gastrointestinal cancer; (2) those aged ≥ 70 years; (3) those signed the informed consent and agreed to participate in this study. The exclusion criteria included: (1) those performed a palliative or emergency operation;



For peer review only - http://bmjopen.bmj.com/site/about/guidelines.xhtml

(2) those aged < 70 years; (3) those cannot be assessed by NRS 2002, MUST and MNA-SF for the difficulty of data collection; (4) those refused to take part in this study.

This study was approved by the ethics committees of the Tenth Affiliated Hospital of Tongji University and the First Affiliated Hospital of Wenzhou Medical University.

## Data collection

The general and anthropometric data of the patients were collected. The general data contained the parameters of age, sex, diagnosis, morbidity, the change of appetite and physical activity. The anthropometric parameters included weight, height, unintentional weight loss, and body mass index (BMI).

## Reference standard: the new ESPEN diagnostic criteria for malnutrition

According to the new ESPEN diagnostic criteria [16], malnutrition was diagnosed when the patients met one of the following two options. Option one required BMI < 18.5 $kg/m^2$. Option two required unintentional weight loss > 10% indefinite of time or > 5% over the last three months combined with reduced BMI (< 20 $kg/m^2$ in patients younger than 70 years or < 22 $kg/m^2$ in patients older than 70 years).

## Assessment of nutritional risk

The assessment was performed by using the following nutritional



For peer review only - http://bmjopen.bmj.com/site/about/guidelines.xhtml

screening tools: NRS 2002, MUST, and MNA-SF.

NRS 2002 is proposed by the ESPEN guidelines based on an analysis of controlled clinical trials [13]. It is designed to identify who needs nutritional supporting. This tool contains a severity of disease score, a nutritional score and an age score. Severity of disease score: one point for hip fracture, long-term hemodialysis, diabetes mellitus, or chronic disease with acute complications; two points for major abdominal surgery, hematological malignancies, stroke, or severe pneumonia; three points for head injury, bone marrow transplantation, or intensive care patients with APACHE more than 10. Nutritional score: one point for weight loss > 5% in 3 months or food intake 50-75% of the common condition; two points for weight loss > 5% in 2 months or BMI 18.5-20.5 kg/$m^2$ with impaired general condition or food intake 25-60% of the general condition; three points for weight loss > 5% in 1 month or BMI less than 18.5 kg/$m^2$ with impaired general condition or food intake reduced by 25% compared with normal condition. Age score: one point for age ≥ 70 years. Nutritional risk was assessed by summarizing the severity disease score, nutritional score and the age score. Patients with total score < 3 are at no or low risk, and with score ≥ 3 are at high risk.

MUST is developed to assess the nutritional risk for adults [14]. It consists of parameters of BMI, unintentional weight loss and any acute disease which compromises nutritional intake for more than 5 days. The



For peer review only - http://bmjopen.bmj.com/site/about/guidelines.xhtml

three parameters are rated as 0, 1, or 2 as follows: BMI > 20 kg/$m^2$ = 0; 18.5-20 kg/$m^2$ = 1; < 18.5 kg/$m^2$ = 2; unintentional weight loss in the past 3-6 months < 5% = 0; 5-10% = 1; > 10% = 2; acute disease: absent = 0; present = 2. Overall risk of malnutrition is assessed by adding all the points together. 0 is at low risk; score 1 is at medium risk; and score 2 is at high risk.

MNA-SF is the short form of MNA, and it is designed especially for the elderly. It contains six questions selected from MNA.[15] These questions are about BMI, recent weight loss, change of appetite, mobility, psychological stress and neuropsychological problems. Each question is rated from 0 to 2 or 3 and the total score of MNA-SF is 14. Patients with 12-14 points are at normal nutritional status. And patients with scores ≤ 11 are at risk of malnutrition.

## Statistical analysis

Statistical analysis was performed using SPSS software version 23.0 for windows. Normally distributed continuous variables were expressed as mean values and standard deviations (SD), categorical variables were presented as absolute and relative frequencies. Independent t test and the Pearson $\chi^2$ test (or Fisher's exact test) were applied to the appropriate comparison of variables. All reported P-values were compared to a significance level of 5% based on two-sided tests. To determine diagnostic concordance between the three assessment tools and the new



For peer review only - http://bmjopen.bmj.com/site/about/guidelines.xhtml

ESPEN diagnostic criteria for malnutrition, Cohen's kappa (κ) statistic was calculated. K coefficient reflects the consistency for qualitative variables. K = 1 means completely consistency between the variables. And if there is no consistency among the variables then K ≤ 0. Positive likelihood ratios (LR+) and negative likelihood ratios (LR-) were calculated for all three tools.

Sensitivity and specificity values for the three nutritional screening tools with the new ESPEN diagnostic criteria for malnutrition were calculated. Receiver operating characteristic curves (ROC) of the three screening tools were also used to evaluate the ability to accurately distinguish the malnourished patients. Area under the ROC curve (AUC) equal to 0.5 indicates that a tool has no diagnostic value, AUC equal to 0.5-0.7 indicates a tool has a low diagnostic value, AUC equal to 0.7-0.9 indicates a tool has a moderate diagnostic value, and AUC equal to 0.9-1 means a tool has a high diagnostic value.

## Results

Two hundred and fifty five patients were enrolled in this study (103 patients underwent gastric cancer surgery and 152 patients underwent colorectal cancer surgery). The characteristics of the sample were presented in Table 1.

**Table 1. Characteristics of the total patients.**

| Patient Characteristics | Total (n=255) | GC (n=103) | CRC (n=152) | P value |
|---|---|---|---|---|
| Age [a] (years) | 76.5±4.8 | 76.1±4.6 | 76.8±4.9 | 0.318 |
| Sex [b] | | | | |



For peer review only - http://bmjopen.bmj.com/site/about/guidelines.xhtml

| | | | | |
|---|---|---|---|---|
| Male | 160(62.7%) | 78(75.7%) | 82(52.1%) | <0.001* |
| Female | 95(37.3%) | 25(24.3%) | 70(47.9%) | |
| Height [a] (m) | 1.61±0.08 | 1.61±0.07 | 1.60±0.09 | 0.269 |
| Weight [a] (kg) | 59.20±10.73 | 59.67±10.26 | 58.90±11.07 | 0.597 |
| BMI [a] (kg/$m^2$) | 22.93±3.55 | 22.93±3.50 | 22.96±3.59 | 0.994 |
| BMI [b] (＜18.5kg/$m^2$) | 26(10.2%) | 11(10.7%) | 15(9.9%) | 0.834 |
| Weight loss [b] (＞5% in 3 months or ＞10% indefinite of time) | 74(29.0%) | 31(30.1%) | 43(28.3%) | 0.755 |

BMI: body mass index. GC: gastric cancer. CRC: colorectal cancer. *Statistically significance ($p \leq 0.05$).

[a] Values expressed as mean ± standard deviation(SD) .

[b] Values expressed as frequencies and percentages.

P values were determined with the use of independent t test and the Pearson$\chi^2$ test.

Table 2 listed the characteristics and anthropometric data of the patients summarized and stratified according to the nutritional status. There were no difference in age or sex between the two groups classified by the three screening tools and the new ESPEN criteria for malnutrition. However, the BMI and weight loss (> 5% in 3 months or > 10% indefinite of time) differed between the groups.



For peer review only - http://bmjopen.bmj.com/site/about/guidelines.xhtml

**Table 2. Characteristics and anthropometric data stratified by the nutrition status of total patients.**

| | ESPEN criteria | | | NRS 2002 | | | MUST | | | MNA-SF | | |
|---|---|---|---|---|---|---|---|---|---|---|---|---|
| | Not malnourished (n=204) | Malnourished (n=51) | P value | No or Low risk (n=122) | High risk (n=133) | P value | Low risk (n=159) | Moderate/High risk (n=96) | P value | No risk (n=133) | Risk of malnutrition (n=122) | P value |
| Age [a] (years) | 76.3±4.8 | 77.4±4.9 | 0.148 | 75.9±4.5 | 77.0±5.0 | 0.059 | 76.1±4.6 | 77.1±5.1 | 0.097 | 76.0±4.6 | 77.0±5.0 | 0.102 |
| Sex [b] | | | | | | | | | | | | |
| male | 124(60.8%) | 36(70.6%) | 0.195 | 73(59.8%) | 87(65.4%) | 0.357 | 101(63.5%) | 59(61.5%) | 0.741 | 90(67.7%) | 70(57.4%) | 0.089 |
| female | 80(39.2%) | 15(29.4%) | | 49(40.2%) | 46(34.6%) | | 58(36.5%) | 37(38.5%) | | 43(32.3%) | 52(42.6%) | |
| Height [a] (m) | 1.60±0.08 | 1.63±0.08 | 0.046* | 1.60±0.08 | 1.61±0.09 | 0.155 | 1.60±0.08 | 1.61±0.09 | 0.511 | 1.61±0.08 | 1.60±0.08 | 0.112 |
| Weight [a] (kg) | 61.55±10.00 | 49.80±8.17 | ＜0.001* | 60.23±10.04 | 58.26±11.29 | 0.142 | 62.46±9.55 | 53.80±10.45 | ＜0.001* | 63.32±9.56 | 54.72±10.16 | ＜0.001* |
| BMI [a] ($kg/m^2$) | 23.98±3.01 | 18.76±2.20 | ＜0.001* | 23.56±3.27 | 22.36±3.70 | 0.006* | 24.28±2.95 | 20.71±3.34 | ＜0.001* | 24.30±2.98 | 21.45±3.52 | ＜0.001* |
| BMI [b] (＜18.5$kg/m^2$) | 1(0.5%) | 25(49.0%) | ＜0.001* | 4(3.3%) | 22(16.5%) | 0.001* | 1(0.6%) | 25(26.0%) | ＜0.001* | 1(0.8%) | 25(20.5%) | ＜0.001* |
| Weight loss [b] (＞5% in 3 months or ＞10% indefinite of time) | 34(16.7%) | 40(78.4%) | ＜0.001* | 4(3.3%) | 70(52.6%) | ＜0.001* | 14(8.8%) | 60(62.5%) | ＜0.001* | 4(3.0%) | 70(57.4%) | ＜0.001* |

*Statistically significance ($p \leq 0.05$).

[a] Values expressed as mean ± standard deviation(SD) .

[b] Values expressed as frequencies and percentages.

P values were determined with the use of independent t test and the Pearson $\chi^2$ test(or Fisher's exact test where appropriate). BMI: body mass index.

Among the patients, the prevalence of malnutrition was 20.0% when determined by the ESPEN criteria. Among patients who underwent curative gastrectomy, the prevalence of malnutrition was 22.3% when determined by the new ESPEN criteria. And among colorectal surgery patients, the prevalence of malnutrition was 18.4% when determined by the



For peer review only

For peer review only - http://bmjopen.bmj.com/site/about/guidelines.xhtml

new ESPEN criteria. The classification of nutritional risk according to the three screening tools were shown in Table 3.

**Table 3. Classification of the risk of malnutrition with the ESPEN criteria and the three screening tools.**

| Risk of malnutrition | Total | | | GC (n=103) | | | CRC (n=152) | | |
|---|---|---|---|---|---|---|---|---|---|
| | NRS 2002 | MUST | MNA-SF | NRS 2002 | MUST | MNA-SF | NRS 2002 | MUST | MNA-SF |
| No/Low | 47.8% | 62.4% | 52.2% | 47.6% | 54.4% | 56.3% | 48.0% | 67.8% | 49.3% |
| | (122/255) | (159/255) | (133/255) | (49/103) | (56/103) | (58/103) | (73/152) | (103/152) | (75/152) |
| Moderate/High | 52.2% | 37.6% | 47.8% | 52.4% | 45.6% | 43.7% | 52.0% | 32.2% | 50.7% |
| | (133/255) | (96/255) | (122/255) | (54/103) | (47/103) | (45/103) | (79/152) | (49/152) | (77/152) |

GC: gastric cancer. CRC: colorectal cancer.

a Classification of malnutrition according to the ESPEN consensus definition of malnutrition.

Cross tabulation of the results of the three tools and the classification of malnutrition according to the ESPEN consensus definition of malnutrition can be found in Table 4.

**Table 4. Cross tabulation of the results of the three screening tools and the classification of malnutrition according to the ESPEN consensus definition of malnutrition.**

| | | NRS 2002 | | MUST | | MNA-SF | |
|---|---|---|---|---|---|---|---|
| | | No/Low risk | Moderate/High risk | Low risk | Moderate/High risk | No risk | Risk |
| ESPEN criteria | Not Malnourished | 118 | 86 | 156 | 48 | 130 | 74 |
| | Malnourished | 4 | 47 | 3 | 48 | 3 | 48 |



For peer review only - http://bmjopen.bmj.com/site/about/guidelines.xhtml

For peer review only

The consistency between the nutritional screening tools and the new ESPEN diagnostic criteria for malnutrition was different. Among the total patients, MUST and MNA had the same sensitivity (94.1%), and NRS 2002 had the lowest sensitivity (92.2%). Moreover, MUST had the highest specificity (76.5%) compared with NRS 2002 (57.8%) and MNA-SF (63.7%). MUST had the highest positive predictive value (50.0%) and the highest negative predictive value (98.1%).

In the total patients, MUST had the highest K value (K = 0.530, p<0.001) compared with MNA-SF (K = 0.380, p<0.001) and NRS 2002 (K = 0.312, p<0.001). In the gastric group, MUST had the highest K value. In the colorectal group, MUST had a higher level of consistency (K = 0.576, p<0.001) compared with the fair consistencies in NRS 2002 (K = 0.243, p<0.001) and MNA-SF (K = 0.361, p<0.001). Finally, the area under the curve (AUC) calculated by the ROC indicated that all three screening tools had a moderate level of diagnostic value to distinguish a malnourished patient (AUC of NRS 2002, MUST and MNA-SF were found to be 0.750, 0.853 and 0.789, respectively). Results are presented in details in Table 5.

**Table 5. Statistical evaluation of the malnutrition screening tools compared with the diagnostic criteria of the ESPEN consensus.**

| | Total | | | GC | | | CRC | | |
|---|---|---|---|---|---|---|---|---|---|
| | NRS 2002 | MUST | MNA-SF | NRS 2002 | MUST | MNA-SF | NRS 2002 | MUST | MNA-SF |
| Sensitivity (%) | 92.2 | 94.1 | 94.1 | 100.0 | 95.7 | 87.0 | 85.7 | 92.9 | 100.0 |



For peer review only - http://bmjopen.bmj.com/site/about/guidelines.xhtml

| | | | | | | | | | |
|---|---|---|---|---|---|---|---|---|---|
| Specificity (%) | 57.8 | 76.5 | 63.7 | 61.3 | 68.8 | 68.8 | 55.6 | 81.5 | 60.5 |
| Positive predictive value (%) | 35.3 | 50.0 | 39.3 | 42.6 | 46.8 | 44.4 | 30.4 | 53.1 | 36.4 |
| Negative predictive value (%) | 96.7 | 98.1 | 97.7 | 100.0 | 98.2 | 94.8 | 94.5 | 98.1 | 100.0 |
| Positive likelihood ratio (LR+) | 2.18 | 4.00 | 2.59 | 2.61 | 3.06 | 2.78 | 1.93 | 5.02 | 2.53 |
| Negative likelihood ratio (LR-) | 0.13 | 0.08 | 0.09 | 0.00 | 0.06 | 0.19 | 0.26 | 0.09 | 0.00 |
| K value (p) | 0.312(＜0.001) | 0.530(＜0.001) | 0.380(＜0.001) | 0.414(＜0.001) | 0.469(＜0.001) | 0.415(＜0.001) | 0.243(＜0.001) | 0.576(＜0.001) | 0.361(＜0.001) |
| AUC | 0.750 | 0.853 | 0.789 | 0.806 | 0.822 | 0.779 | 0.707 | 0.872 | 0.802 |

GC: gastric cancer. CRC: colorectal cancer.

K value derived from Cohen kappa statistics.

AUC: Area under the curve from ROC.

For peer review only



For peer review only - http://bmjopen.bmj.com/site/about/guidelines.xhtml

# Discussion

This study is the first to apply the new ESPEN diagnostic criteria to the specific population of elderly gastrointestinal cancer patients, and 20.0% of the patients were classified as malnourished according to the new ESPEN diagnostic criteria for malnutrition. Another previous study has investigated the prevalence of malnutrition diagnosed by the new ESPEN criteria in four diverse populations.[19] In that study, 0.5% of the healthy elderly individuals and 6% of the geriatric outpatients were identified as malnourished. It is significantly lower compared with that of our study, which indicated that patients with gastrointestinal cancer might have a higher prevalence of malnutrition. The difference in the malnutrition rates also indicated that we should put emphasis on the requirement to assess the nutritional risk of the hospitalized elderly gastrointestinal cancer patients.

In the present study, 52.2% and 37.6% of the patients were found to be at moderate or high risk of malnutrition according to NRS 2002 and MUST respectively. With the MNA-SF, 47.8% of the patients were found to be at a risk of malnutrition. The various prevalence of the risk of malnutrition can result from the differences between the nutritional screening tools. In our study, MUST had the greatest K value compared with the NRS 2002 and MNA-SF. It showed that a greater proportion of the elderly patients with gastrointestinal cancer who were distinguished to



For peer review only - http://bmjopen.bmj.com/site/about/guidelines.xhtml

be at moderate or high risk of malnutrition with MUST could be identified as malnourished according to the ESPEN diagnostic criteria. In other words, MUST can perform the best to detect the specific malnourished individuals diagnosed by the new ESPEN criteria, compared with NRS 2002 and MNA-SF. Furthermore, MUST has the greatest AUC compared with NRS 2002 and MNA-SF in our study.

Many previous studies compared the three nutritional screening tools in specific populations. Poulia et al. evaluated the efficacy of six nutritional screening tools in the elderly.[20] In Poulia' s study, NRS 2002 was found to overestimate nutritional risk, MNA-SF was proven to have a great validity, and MUST was found to have the best validity and the greatest consistency. Another study by Myoungha et al.[21] evaluated five nutritional screening tools, and suggested that MNA-SF overestimated the nutritional risk in the elderly, and NRS 2002 performed better than MNA-SF. However, MUST was also found to be the most efficient and useful screening tool in this study. Both the previous studies compared the screening tools with a combined index suggested by Pablo et al.,[22] and they confirmed our results for the best performance of MUST compared with NRS 2002 and MNA-SF. While Donini et al. developed a study for the nutritional evaluation of the elderly nursing home residents[23] and found that MNA-SF presented a higher predictive value compared with NRS 2002 and MUST. However, in the study by Donini et



For peer review only - http://bmjopen.bmj.com/site/about/guidelines.xhtml

al., MNA was taken as the reference standard, which might induce an underpowered result. A previous study used the new ESPEN diagnostic criteria for malnutrition as a reference standard to compare nutritional screening tools.[24] However, MNA-SF was not included in this study, and the participators were not merely the elderly.

The results of the comparison among NRS 2002, MUST and MNA-SF showed different efficiency of the three screening tools in our study. There might be an explanation for the differences. The original designs of the three nutritional screening tools were different. NRS 2002 was developed to determine who needs nutritional supporting and it might distinguish an increased number of patients to be at high risk of malnutrition. According to the diagnosis criteria of NRS 2002, 1 score was added to patients aged ≥ 70 years, it might contribute to the higher prevalence of patients at nutritional risk diagnosed by NRS 2002. However, MUST was a screening tool to identify adults who are at risk of malnutrition. MNA-SF, the short form of MNA, was developed as a quick and easy nutritional screening tool and was used for primary screening before further assessment. The different results between our study and others can be attributed to the different populations and reference standards, just as what Ma et al. had mentioned in their review.[25]

Moreover, in our study, we evaluated the adequacy of the nutritional screening tools in the gastric cancer patients and the colorectal cancer



For peer review only - http://bmjopen.bmj.com/site/about/guidelines.xhtml

patients, respectively. In the gastric cancer population, NRS 2002, MUST and MNA-SF all had a same moderate level of consistency with the new ESPEN diagnostic criteria for malnutrition (K = 0.414 for NRS 2002, 0.469 for MUST and 0.415 for MNA-SF, respectively). While in the colorectal population, MUST had the highest level of consistency (K = 0.576) compared with the fair level of consistency of NRS 2002 (K = 0.243) and MNA-SF (K = 0.361). Based on the result, we concluded that MUST could perform the best in the specific colorectal cancer patients. Further studies are needed to confirm our conclusion.

It is of great significance to improve the nutritional status of the elderly patients with gastrointestinal cancer if they are malnourished or at risk of malnutrition. As the first step to identify who are malnourished or at risk of malnutrition, nutritional screening should be reliable and easy to perform. To the best of our knowledge, this is the first study to compare the three nutritional screening tools in the specific geriatric gastrointestinal cancer population. Our study suggested that MUST is the best choice in the three common nutritional screening tools. For the accuracy and simplicity of MUST, both the patients and surgeons will benefit from it. Furthermore, in our study, though there were some differences in sensitivity and specificity, the three tools were found to have a same level of consistency in the gastric cancer population. While in the colorectal cancer population, MUST had the highest level of



For peer review only - http://bmjopen.bmj.com/site/about/guidelines.xhtml

consistency compared with the others(Κ = 0.576). MUST also had the significant highest specificity, positive predictive value, and the greatest AUC. It indicated that we can choose one of three nutritional screening tools when assessing nutritional risk among the elderly patients with gastric cancer. However, when assessing nutritional risk among the elderly patients with colorectal cancer, MUST is the only choice for the highest accuracy.

The ESPEN guideline used to promote NRS 2002 as a tool to screen hospitalized patients. While with the results of this study, MUST was found to be better to screen gastrointestinal cancer patients. This may result from several aspects. MUST was developed as a valid tool to identify nutritional risk of specific patients population, and it is the unique tool that was designed for screening of malnutrition.[26] Therefore, MUST might perform best according to the ESPEN diagnostic criteria for malnutrition. Moreover, MUST has straight forward and objective questions, with which is easier to be performed. It can be a useful nutritional screening tool when there is no redundant time and no professional medical staff.

It is well-known that malnourished patients or patients at risk of malnutrition would have a poor clinical outcome. Therefore, it is of great significance to improve the nutritional status of these patients. The indices of these three screening tools have common characteristics. With



For peer review only - http://bmjopen.bmj.com/site/about/guidelines.xhtml

the results of our study, the parameters of MUST suggest that we could intervene malnutrition by improving BMI, avoiding weight loss and curing the acute disease. In fact, this is partly similar with the parameters of EPSEN diagnostic criteria, NRS 2002 and MNA-SF. It means that if the result of MUST was improved, the consequence of EPSEN diagnostic criteria and other screening tools would also be improved. Further studies should be developed to investigate implications of the intervention for malnutrition.

The present study has some limitations. Firstly, the sample size is relatively small. However, this study was conducted in two centers with large surgical volume. So we believe that the data in our study are more representative. To a certain extent, it overcame the smallness of the sample size. Secondly, according to the new ESPEN diagnostic criteria for malnutrition, malnutrition can also be diagnosed by unintentional weight loss combined with reduced fat free mass index (FFMI). So another limitation of our study is the lack of data for fat free mass index. However, the measurement of FFMI requires specific equipment and extra costs. Moreover, Trummer et al. found that low BMI and low FFMI were closely correlative.[27] In that study, FFMI less than 17 $kg/m^2$ for men and less than 15 $kg/m^2$ for womFen were roughly equivalent to BMI less than 18.5 $kg/m^2$ after determining the FFM levels.

## Conclusions



For peer review only - http://bmjopen.bmj.com/site/about/guidelines.xhtml

To our knowledge, this is the first study to compare the three malnutrition screening tools (NRS 2002, MUST and MNA-SF) with the new ESPEN diagnostic criteria for malnutrition, and it is also the first study to evaluate the three screening tools in the specific geriatric gastrointestinal cancer patients. The prevalence of malnutrition was 20.0% with the ESPEN diagnostic criteria for malnutrition for patients with gastrointestinal cancer in the present study. MUST was found to perform the best to identify the malnourished elderly gastrointestinal cancer patients distinguished by the new ESPEN diagnostic criteria for malnutrition. Nevertheless, further studies are needed to verify our findings.

## References

1. Hurria A, Gupta S, Zauderer M, et al. Developing a cancer-specific geriatric assessment: a feasibility study. Cancer 2005;104:1998-2005. doi:10.1002/cncr.21422.
2. Balducci L, Extermann M. Cancer and aging. An evolving panorama. Hematology/oncology Clinics of North America 2000;14:1-16.
3. Marugame T, Dongmei Q. Comparison of Time Trends in Stomach Cancer Incidence (1973–1997) in East Asia, Europe and USA, from Cancer Incidence in Five Continents Vol. IV–VIII. Jpn J Clin Oncol. 2007;37:242-3. doi:10.1093/jjco/hym014.



For peer review only - http://bmjopen.bmj.com/site/about/guidelines.xhtml

4. Biondi A, Vacante M, Ambrosino I, et al. Role of surgery for colorectal cancer in the elderly. World Journal of Gastrointestinal Surgery 2011;8:606-13. doi:10.4240/wjgs.v8.i9.606
5. Takeshita H, Ichikawa D, Komatsu S, et al. Surgical outcomes of gastrectomy for elderly patients with gastric cancer. World Journal of Surgery 2013;37:2891-8. doi: 10.1007/s00268-013-2210-7.
6. Simmonds PD, Best L, George S, et al. Surgery for colorectal cancer in elderly patients: a systematic review. Lancet 2000;356:968-74. doi:http://dx.doi.org/10.1016/S0140-6736(00)02713-6.
7. Heriot AG, Tekkis PP, Smith JJ, et al. Prediction of postoperative mortality in elderly patients with colorectal cancer. Diseases of the Colon & Rectum 2006;49:816-24. doi: 10.1007/s10350-006-0523-4.
8. Audisio RA, Balch CM. Why Can't Surgeons Treat Older Patients the Same as Younger Patients? Annals of Surgical Oncology 2016;23:4123-5. doi:10.1245/s10434-016-5459-x.
9. Brownie S. Why are elderly individuals at risk of nutritional deficiency? International Journal of Nursing Practice 2006, 12:110-8. doi:10.1111/j.1440-172X.2006.00557.x.
10. Bozzetti F. Why the oncologist should consider the nutritional status of the elderly cancer patient. Nutrition 2015;31:590-3. doi:10.1016/j.nut.2014.12.005.
11. Martucci RB, Barbosa MV, D'Almeida CA, et al. Undernutrition as



For peer review only - http://bmjopen.bmj.com/site/about/guidelines.xhtml

independent predictor of early mortality in elderly cancer patients. Nutrition 2017;34:65-70. doi:10.1016/j.nut.2016.09.011.

12. Kondrup J, Allison SP, Elia M, et al. ESPEN guidelines for nutrition screening 2002. Clinical Nutrition 2003;22:415-21. doi:10.1016/S0261-5614(03)00098-0.
13. Hamberg O. Nutritional risk screening (NRS 2002): a new method based on an analysis of controlled clinical trials. Clinical Nutrition 2003;22:321-36. doi:10.1016/S0261-5614(02)00214-5.
14. Elia M. The 'MUST' report. Nutritional screening for adults: a multidisciplinary responsibility. Development and use of the 'Malnutrition Universal Screening Tool' (MUST) for adults. British Association for Parenteral and Enteral Nutrition (BAPEN) 2003.
15. Rubenstein LZ, Harker JO, Salvà A, et al. Screening for undernutrition in geriatric practice: developing the short-form mini-nutritional assessment (MNA-SF). Journals of Gerontology 2001;56:M366-72.
16. Cederholm T, Bosaeus I, Barazzoni R, et al. Diagnostic criteria for malnutrition - An ESPEN Consensus Statement. Clinical Nutrition 2015;139:335-40. doi:10.1016/j.clnu.2015.03.001.
17. Guerra RS, Fonseca I, Sousa AS, et al. ESPEN diagnostic criteria for malnutrition – A validation study in hospitalized patients. Clinical Nutrition 2016 [Epub ahead of print] doi:10.1016/j.clnu.2016.08.022.



For peer review only - http://bmjopen.bmj.com/site/about/guidelines.xhtml

18. Rondel AL, Langius JA, de van der Schueren MA, et al. The new ESPEN diagnostic criteria for malnutrition predict overall survival in hospitalised patients. Clinical Nutrition 2016 [Epub ahead of print] doi:10.1016/j.clnu.2016.11.018.

19. Rojer AG, Kruizenga HM, Trappenburg MC, et al. The prevalence of malnutrition according to the new ESPEN definition in four diverse populations. Clinical Nutrition 2015;35:758-62. doi:10.1016/j.clnu.2015.06.005.

20. Poulia KA, Yannakoulia M, Karageorgou D, et al. Evaluation of the efficacy of six nutritional screening tools to predict malnutrition in the elderly. Clinical Nutrition 2012;31:378-85. doi:10.1016/j.clnu.2011.11.017.

21. Myoungha B, Youngran H. Evaluation of the efficacy of nutritional screening tools to predict malnutrition in the elderly at a geriatric care hospital. Nutrition Research & Practice 2015;9:637-43. doi:10.4162/nrp.2015.9.6.637.

22. Pablo AM, Izaga MA, Alday LA. Assessment of nutritional status on hospital admission: nutritional scores. European Journal of Clinical Nutrition 2003;57:824-31. doi:10.1038/sj.ejcn.1601616.

23. Donini LM, Poggiogalle E, Molfino A, et al. Mini-Nutritional Assessment, Malnutrition Universal Screening Tool, and Nutrition Risk Screening Tool for the Nutritional Evaluation of Older Nursing



For peer review only - http://bmjopen.bmj.com/site/about/guidelines.xhtml

Home Residents. J Am Med Dir Assoc 2016;17:959.e11-8. doi:10.1016/j.jamda.2016.06.028.

24. Poulia KA, Klek S, Doundoulakis I, et al. The two most popular malnutrition screening tools in the light of the new ESPEN consensus definition of the diagnostic criteria for malnutrition. Clinical Nutrition 2017;36:1130-5. doi:10.1016/j.clnu.2016.07.014.

25. van Bokhorst-de van der Schueren MA, Guaitoli PR, Jansma EP, et al. Nutrition screening tools: does one size fit all? A systematic review of screening tools for the hospital setting. Clinical Nutrition 2014;33:39-58. doi:10.1016/j.clnu.2013.04.008.

26. Stratton RJ, Hackston A, Longmore D, et al. Malnutrition in hospital outpatients and inpatients: prevalence, concurrent validity and ease of use of the 'malnutrition universal screening tool' ('MUST') for adults. British Journal of Nutrition 2004;92:799-808.

27. Bahadori B, Uitz E, Tonninger-Bahadori K, et al. Body composition: the fat-free mass index (FFMI) and the body fat mass index (BFMI) distribution among the adult Austrian population-results of a cross-sectional pilot study. International Journal of Body Composition Research 2006;4:123-8.

**Contributors:** Xiao-Jun Ye and Yan-Bin Ji are joint first authors. Zhen Yu, Cheng-Le Zhuang, and Xian Shen designed the study. Bing-Wei Ma



For peer review only - http://bmjopen.bmj.com/site/about/guidelines.xhtml

and Wei-Zhe Chen collected the data. Dong-Dong Huang and Zong-You Pan analyzed the data. Xiao-Jun Ye and Yan-Bin Ji drafted the manuscript.Zhen Yu and Cheng-Le Zhuang approved the final version of the manuscript. All authors have read and approved the final manuscript.

**Data sharing statement:** No additional unpublished data are available.

For peer review only



For peer review only - http://bmjopen.bmj.com/site/about/guidelines.xhtml

| Section & Topic | No. | Item | Page |
|---|---|---|---|
| Title or Abstract | | | |
| | 1 | Identification as a study of diagnostic accuracy using at least one measure of accuracy (such as sensitivity, specificity, predictive values, or AUC) | 3 |
| Abstract | | | |
| | 2 | Structured summary of study design, methods, results, and conclusions (for specific guidance, see STARD for Abstracts) | 3 |
| Introduction | | | |
| | 3 | Scientific and clinical background, including the intended use and clinical role of the index test | 5 |
| | 4 | Study objectives and hypotheses | 6 |
| Methods | | | |
| Study Design | 5 | Whether data collection was planned before the index test and reference standard were performed (prospective study) or after (retrospective study) | 6 |
| Participants | 6 | Eligibility criteria | 6 |
| | 7 | On what basis potentially eligible participants were identified (such as symptoms, results from previous tests, inclusion in registry) | 6 |
| | 8 | Where and when potentially eligible participants were identified (setting, location and dates) | 6 |
| | 9 | Whether participants formed a consecutive, random or convenience series | 6 |
| Test method | 10a | Index test, in sufficient detail to allow replication | 7,8,9, 10 |
| | 10b | Reference standard, in sufficient detail to allow replication | 7,8,9, 10 |
| | 11 | Rationale for choosing the reference standard (if alternatives exist) | 7,8,9, 10 |
| | 12a | Definition of and rationale for test positivity cut-offs or result categories of the index test, distinguishing pre-specified from exploratory | 7,8,9, 10 |
| | 12b | Definition of and rationale for test positivity cut-offs or result categories of the reference standard, distinguishing pre-specified from exploratory | 7,8,9, 10 |
| | 13a | Whether clinical information and reference standard results were available to the performers/readers of the index test | 7 |
| | 13b | Whether clinical information and index test results were available to the assessors of the reference standard | 7 |
| Analysis | 14 | Methods for estimating or comparing measures of diagnostic accuracy | 9,10 |
| | 15 | How indeterminate index test or reference standard results were handled | 9,10 |
| | 16 | How missing data on the index test and reference standard were handled | 6 |
| | 17 | Any analyses of variability in diagnostic accuracy, distinguishing pre-specified from exploratory | 9,10 |
| | 18 | Intended sample size and how it was determined | 6 |
| Results | | | |
| Participants | 19 | Flow of participants, using a diagram | 10 |
| | 20 | Baseline demographic and clinical characteristics of participants | 10 |
| | 21a | Distribution of severity of disease in those with the target condition | 12 |
| | 21b | Distribution of alternative diagnoses in those without the target condition | 12 |
| | 22 | Time interval and any clinical interventions between index test and reference standard | 13 |
| The Results | 23 | Cross tabulation of the index test results (or their distribution) by the results of the reference standard | 14 |
| | 24 | Estimates of diagnostic accuracy and their precision (such as 95% confidence intervals) | 15 |
| | 25 | Any adverse events from performing the index test or the reference standard | 15 |
| Discussion | | | |
| | 26 | Study limitations, including sources of potential bias, statistical uncertainty, and generalisability | 21 |

For peer review only

| | | | |
|---|---|---|---|
| | 27 | Implications for practice, including the intended use and clinical role of the index test | 19,20 |
| Other Information | | | |
| | 28 | Registration number and name of registry | 2 |
| | 29 | Where the full study protocol can be accessed | 6 |
| | 30 | Sources of funding and other support; role of funders | 2 |

For peer review only
